# Using Character-Level and Entity-Level Representations to Enhance Bidirectional Encoder Representation From Transformers-Based Clinical Semantic Textual Similarity Model: ClinicalSTS Modeling Study

**DOI:** 10.2196/23357

**Published:** 2020-12-29

**Authors:** Ying Xiong, Shuai Chen, Qingcai Chen, Jun Yan, Buzhou Tang

**Affiliations:** 1 Harbin Institute of Technology Shenzhen China; 2 Peng Cheng Laboratory Shenzhen China; 3 Yidu Cloud Technology Company Limited Beijing China

**Keywords:** natural language processing, deep learning, clinical semantic textual similarity, knowledge graph

## Abstract

**Background:**

With the popularity of electronic health records (EHRs), the quality of health care has been improved. However, there are also some problems caused by EHRs, such as the growing use of copy-and-paste and templates, resulting in EHRs of low quality in content. In order to minimize data redundancy in different documents, Harvard Medical School and Mayo Clinic organized a national natural language processing (NLP) clinical challenge (n2c2) on clinical semantic textual similarity (ClinicalSTS) in 2019. The task of this challenge is to compute the semantic similarity among clinical text snippets.

**Objective:**

In this study, we aim to investigate novel methods to model ClinicalSTS and analyze the results.

**Methods:**

We propose a semantically enhanced text matching model for the 2019 n2c2/Open Health NLP (OHNLP) challenge on ClinicalSTS. The model includes 3 representation modules to encode clinical text snippet pairs at different levels: (1) character-level representation module based on convolutional neural network (CNN) to tackle the out-of-vocabulary problem in NLP; (2) sentence-level representation module that adopts a pretrained language model bidirectional encoder representation from transformers (BERT) to encode clinical text snippet pairs; and (3) entity-level representation module to model clinical entity information in clinical text snippets. In the case of entity-level representation, we compare 2 methods. One encodes entities by the entity-type label sequence corresponding to text snippet (called entity I), whereas the other encodes entities by their representation in MeSH, a knowledge graph in the medical domain (called entity II).

**Results:**

We conduct experiments on the ClinicalSTS corpus of the 2019 n2c2/OHNLP challenge for model performance evaluation. The model only using BERT for text snippet pair encoding achieved a Pearson correlation coefficient (PCC) of 0.848. When character-level representation and entity-level representation are individually added into our model, the PCC increased to 0.857 and 0.854 (entity I)/0.859 (entity II), respectively. When both character-level representation and entity-level representation are added into our model, the PCC further increased to 0.861 (entity I) and 0.868 (entity II).

**Conclusions:**

Experimental results show that both character-level information and entity-level information can effectively enhance the BERT-based STS model.

## Introduction

### Background

Electronic health record (EHR) systems have been widely used in hospitals all over the world for convenience to health information storage, share, and exchange [1]. In recent years, EHRs have become a key data source for medical research and clinical decision support. Therefore, the quality of EHRs is crucial. However, copy-and-paste and templates are very common in EHR writing [2,3], resulting in EHRs of low quality in content. How to detect copy-and-paste and templates in different documents has become increasingly important for the secondary use of EHRs. This can be regarded as a clinical semantic textual similarity (ClinicalSTS) task, which is also applied to clinical decision support, trial recruitment, tailored care, clinical research [4-6], and medical information services, such as clinical question answering [7,8] and document classification [9].

In the past few years, some shared tasks on STS, such as Semantic Evaluation (SemEval), have been launched by different organizers [10-14]. These shared tasks mainly focus on general domains, including newswire, tutorial dialog system, Wikipedia, among others. There has been almost no study on STS in the clinical domain. To boost the development of ClinicalSTS, Wang et al [15] constructed a clinical STS corpus of 174,629 clinical text snippet pairs from Mayo Clinic. Based on a part of this corpus, BioCreative/OHNLP organizers held the first ClinicalSTS shared pilot task (challenge) in 2018 [16]. A corpus of 1068 clinical text snippet pairs with similarity ranging from 0 to 5 was provided for this shared task. In 2019, the n2c2/OHNLP organizers extended the 2018 shared task corpus and continued to hold ClinicalSTS shared task [17]. The extended corpus is composed of 2055 clinical text snippet pairs.

In this paper, we introduce our system developed for the 2019 n2c2/OHNLP shared task on ClinicalSTS. The system is based on bidirectional encoder representation from transformers (BERT) [18] and includes the 2 other types of representations besides BERT: (1) character-level representation to tackle the out-of-vocabulary (OOV) problem in natural language processing (NLP) and (2) entity-level representation to model clinical entity information in clinical text snippets. In the case of entity-level representation, we apply 2 entity-level representations: one encodes entities in a text snippet by the corresponding entity label sequence (called entity I) and the other one encodes entities with their representation on Mesh [19] (called entity II). Our system achieves the highest Pearson correlation coefficient (PCC) of 0.868 on the corpus of the 2019 n2c2/OHNLP track on ClinicalSTS, which is competitive with other state-of-the-art systems.

### Related Work

A model for STS usually consists of 2 modules: a module to encode text snippet (or sentence) pairs and a module for prediction (classification or regression). According to sentence pair encoding, STS models can be classified into the following 2 categories: sentence encoding models and sentence pair interaction models. The sentence encoding models first use Siamese neural network to individually encode 2 sentences with 2 neural networks of the same structure and shared parameters [20-23], then combine the 2 sentences’ representation through concatenation, element-wise product, or element-wise difference operations, and finally make a classification or regression prediction via a specific layer such as multilayer perceptron (MLP) [24]. The main limitation of the sentence pair encoding models is that they ignore word-level interactions. The sentence pair interaction models adopt matching-aggregation architectures to encode word-level interactions [25,26]. These models first build an interaction matrix and then use a convolutional neural network (CNN) [27] and long short-term memory [28] with attention mechanism [29,30] and hierarchical architecture [31] to obtain aggregated matching representation for final prediction.

In recent years, pretrained language models good at capturing sentence-level semantic information, such as BERT [18], XLNet [32], RoBERTa [33], have been proved to significantly improve downstream tasks. However, most pretrained language models are at the token level. In order to tackle the inherent OOV problem of NLP, character-level representation is also considered in various NLP tasks, such as named entity recognition [34-36] and entity normalization [37], and brings improvements. Besides, researchers have started investigating how to use entity-level representation in NLP tasks [38,39].

## Methods

### Data Set

The n2c2/OHNLP organizers manually annotated a total of 2055 clinical text snippet pairs by 2 medical experts for the ClinicalSTS task, where 1643 pairs are used as the training set and 412 as the test set. The similarity of each clinical text snippet pair is measured by PCC ranging from 0 to 5, where 0 means that 2 clinical text snippets are absolutely different, and 5 means that 2 clinical text snippets are entirely semantically equal. All clinical text snippets are selected from deidentified EHRs. Table 1 gives examples of each score.

**Table 1:** Examples of ClinicalSTS^a^.

Score	Example of clinical text snippet pair	
0	**The 2 sentences are completely dissimilar**	
		S1: The patient has missed 0 hours of work in the past seven days for issues not related to depression.
		S2: In the past year the patient has the following number of visits: none in the hospital none in the er and one as an outpatient.
1	**The 2 sentences are not equivalent but have the same topic**	
		S1: There is no lower extremity edema present bilaterally.
		S2: There is a 2+ radial pulse present in the upper extremities bilaterally.
2	**The 2 sentences are not equivalent but share some details**	
		S1: I met with the charge nurse and reviewed the patient's clinical condition.
		S2: I have reviewed the relevant imaging and medical record.
3	**The 2 sentences are roughly equivalent but some important information differs**	
		S1: I explained the diagnosis and treatment plan in detail, and the patient clearly expressed understanding of the content reviewed.
		S2: Began discussion of diagnosis and treatment of chronic pain and chronic fatigue; patient expressed understanding of the content.
4	**The 2 sentences are mostly equivalent and only a little detail is different**	
		S1: Albuterol [PROVENTIL/VENTOLIN] 90 mcg/Act HFA Aerosol 2 puffs by inhalation every 4 hours as needed.
		S2: Albuterol [PROVENTIL/VENTOLIN] 90 mcg/Act HFA Aerosol 1-2 puffs by inhalation every 4 hours as needed #1 each.
5	**The 2 sentences mean the same thing, they are absolutely equivalent**	
		S1: Goals/Outcomes: Patient will be instructed in a home program, demonstrate understanding, and state the ability to continue independently.
		S2: Patient will be instructed in home program, demonstrate understanding, and state ability to continue independently-ongoing.

^a^ClinicalSTS: clinical semantic textual similarity.

### Models

**Figure 1:**
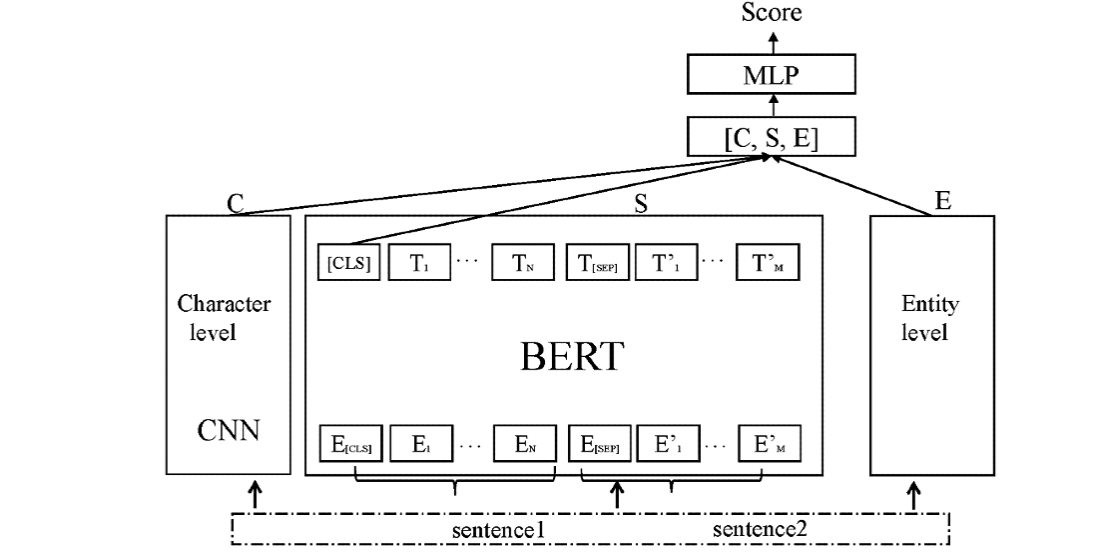
Overview architecture of our model for the ClinicalSTS track of the 2019 n2c2/OHNLP challenge. BERT: bidirectional encoder representation from transformers; ClinicalSTS: clinical semantic textual similarity; CNN: convolutional neural network; MLP: multilayer perceptron; PCC: Pearson correlation coefficient; [CLS]: the representation of sentence pair with BERT.

Figure 1 presents an overview architecture of our model. In this model, we first use 3 representation modules at different levels to encode input text snippet pairs, that is, character-level, sentence-level, and entity-level representation modules, and then feed them to MLP for prediction.

#### Character-Level Representation

In order to tackle the OOV problem in NLP, following [34-37], given a pair of clinical text snippets (a, b), we first apply character-level CNN on each token to obtain its character-level representation, and then apply max pooling operation on all tokens in a and b to obtain the character-level representation of (a, b), denoted by C. We model the character-level representation with CNN, because there is no significant difference in using CNN and long short-term memory, according to previous studies [40,41].

#### Sentence-Level Representation

We use BERT to encode the input clinical text snippet pair (a, b) and obtain its sentence-level representation, denoted by S = BERT(a, b).

#### Entity-Level Representation

**Figure 2:**
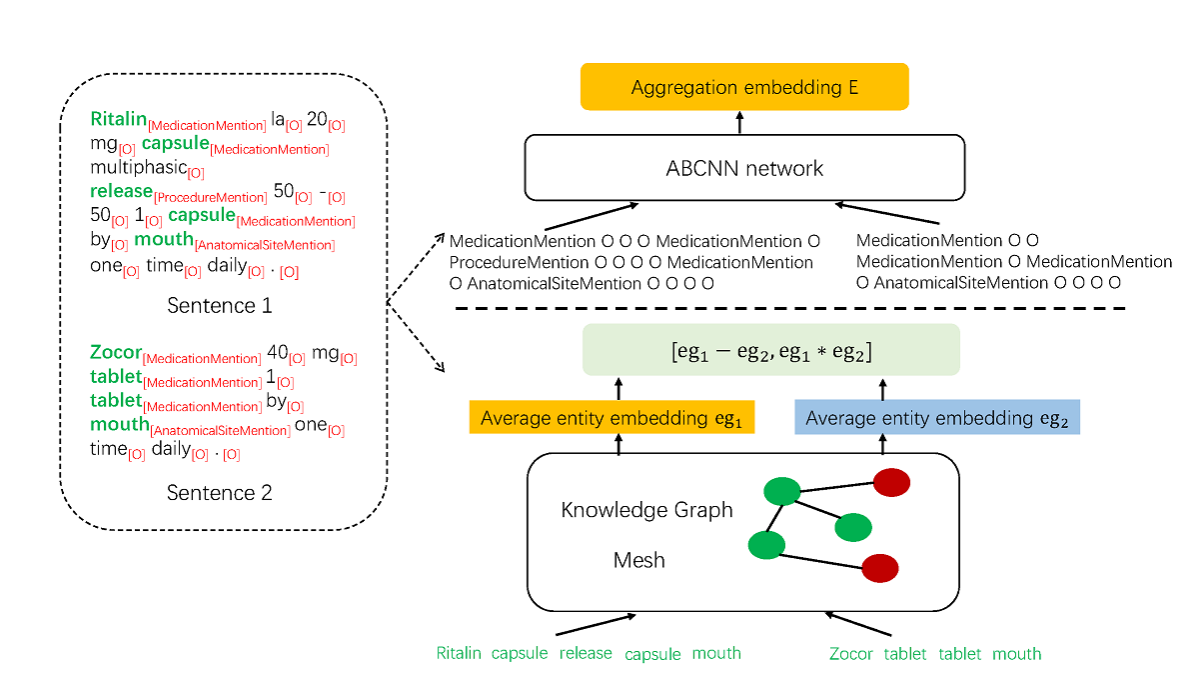
Entity-level representation.

We first deploy cTAKES [42], a popular clinical NLP tool, to extract entity mentions from text snippets, and then propose 2 methods to obtain the entity-level representations of the text snippets according to the extracted entity mentions, as shown in Figure 2. cTAKES can extract 9 kinds of entities: AnatomicalSiteMention, DiseaseDisorderMention, FractionAnnotation, MedicationMention, Predicate, ProcedureMention, RomanNumeralAnnotation, SignSymptomMention, and Temporal Information.

In the first method for entity-level representation (entity I), we convert text snippet a and b into entity-type sequences corresponding to them, and then deploy attention-based CNN [27] on the pair of the entity-type sequences in the following way:

E = BCNN(es_a_, es_b_) (1)

where es_a_ is the entity label sequence of text snippet a, es_b_ is the entity label sequence of text snippet b, BCNN is basic bi-CNN, and E is the entity-level representation of (es_a_, es_b_). For example, given a text snippet b “Zocor 40 mg tablet 1 tablet by mouth one time daily.” shown in Figure 2, cTAKES first extracts 3 medication mentions {“Zocor”, “tablet”, “tablet”} and 1 anatomical mention {“mouth”}, and then we obtain the entity-type sequence corresponding to text snippet b: “MedicationMetion O O MedicationMetion O MedicationMetion O AnatomicalSiteMention O O O O”. In this entity-type sequence, “O” stands for “Other.”

The second method for entity-level representation (entity II) first directly adopts entity representation learned by TransE [43] on an external knowledge graph (KG; Mesh in this study), and then applies average pooling operation on all entities individually in sentences a and b to get entity-level representations of a (denoted by eg_a_) and b (denoted by eg_b_) respectively, and finally aggregates their representations using equation 2.

E = tanh (W_e_[eg_a_ – eg_b_; eg_a_ * eg_b_] + b_e_) (2)

where “[;]” denotes concatenation operation, W_e_ is a weight matrix, and b_e_ is a bias vector.

#### MLP Layer

To aggregate the information of 3 modules, we concatenate them together:

f = [S; C; E] (3)

Then, we use MLP (as shown in equation 4) to predict the STS score p_score_ of (a, b) as follows:

p_score_ = MLP(Wf + b) (4)

where W is a weight matrix, and b is a bias vector.

The loss function used in our model is the minimum square error (MSE) function:

Loss = MSE(p_score_ – g_score_) (5)

where g_score_ is the gold-standard score.

#### Experimental Setting

Before conducting experiments, we preprocess the corpus using the following simple rules: (1) convert clinical text snippets into lowercase; (2) tokenize clinical text snippets using special symbols, such as “[”, “]”, “/”, “,”, and “.”, and keep them unstained in some situations such as “.” in decimals. The hyperparameters of our model are shown in Table 2. Other parameters are optimized via fivefold cross validation on the training set. The pretrained BERT model used for text snippet pair representation in our experiments is [BERT-Base, Uncased] [44]. We train all model parameters simultaneously, set epochs as 12, and save the last checkpoints as the final models. The performance of all models is measured by PCC.

**Table 2:** Hyperparameters setting of our model.

Parameters	Value
Learning rate	2 × 10^–5^
Sequence length of BERT^a^	380
Epochs	12
Batch size	20
Knowledge graph embedding dimension d	100
Character-level kernel size	3
Convolution kernels of BCNN^b^	50
Kernel size of BCNN	3
Word embedding dimension of entity I	50

^a^BERT: bidirectional encoder representation from transformers.

^b^BCNN: Basic bi-CNN.

## Results

Table 3 shows the overall results of our proposed model. Our model achieves the highest PCC of 0.868, which is competitive with other state-of-the-art models proposed for the 2019 n2c2/OHNLP track on ClinicalSTS. The model using entity II is better than that using entity I by 0.007 in PCC, indicating that entity II is a better supplement to BERT than entity I. When character-level representation is removed, the PCC of our model decreases to 0.859 (entity I) and 0.854 (entity II). When entity-level representation is removed, the PCC of our model decreases to 0.858. When both types of representations are removed, the PCC of our model further decreases to 0.848. The results indicate that both character-level representation and entity-level representation are supplementary to BERT. Although the improvements individually from entity I and character-level text snippet representation are more remarkable than entity II, the improvement from the combination of entity I and character-level representation is much smaller than the combination of entity II and character-level representation. It is because both character-level representation and entity I come from text snippets, whereas entity II comes from external KG. The diversity between character-level representation and entity II is much larger than that between character-level representation and entity I. It is interesting that our model is not further improved when both entity I and entity II are considered in our model at the same time, which may be also because of the diversity.

Moreover, we investigate the effect of the domain-specific pretrained BERT models [45,46] on our model. We replace the pretrained BERT model in the general domain, [BERT-Base, Uncased] [44], by the pretrained BERT model in the clinical domain [45] to obtain a new model. The highest PCC of the new model is 0.872, which is slightly better than our previous model, indicating that the domain-specific pretrained BERT model is beneficial to our model.

**Table 3:** Pearson correlation coefficient of our model on the test set.

Model and setting		PCC^a^
**Our model**		
	Entity I	0.861
	Entity II	0.868^b^
	Entity I + Entity II	0.862
**Without** **character** **-level text snippet representation**		
	Entity I	0.859
	Entity II	0.854
Without entity-level representation		0.858
Without both		0.848

^a^PCC: Pearson correlation coefficient.

^b^The highest PCC.

## Discussion

### Error Analysis

Although the proposed model achieves competitive performance, there are also some errors. To analyze these errors, we look into samples for which the difference between the predicted STS score and gold-standard similarity score is greater than 1.0 and find that the main errors can be classified into 2 types.

**Figure 3:**
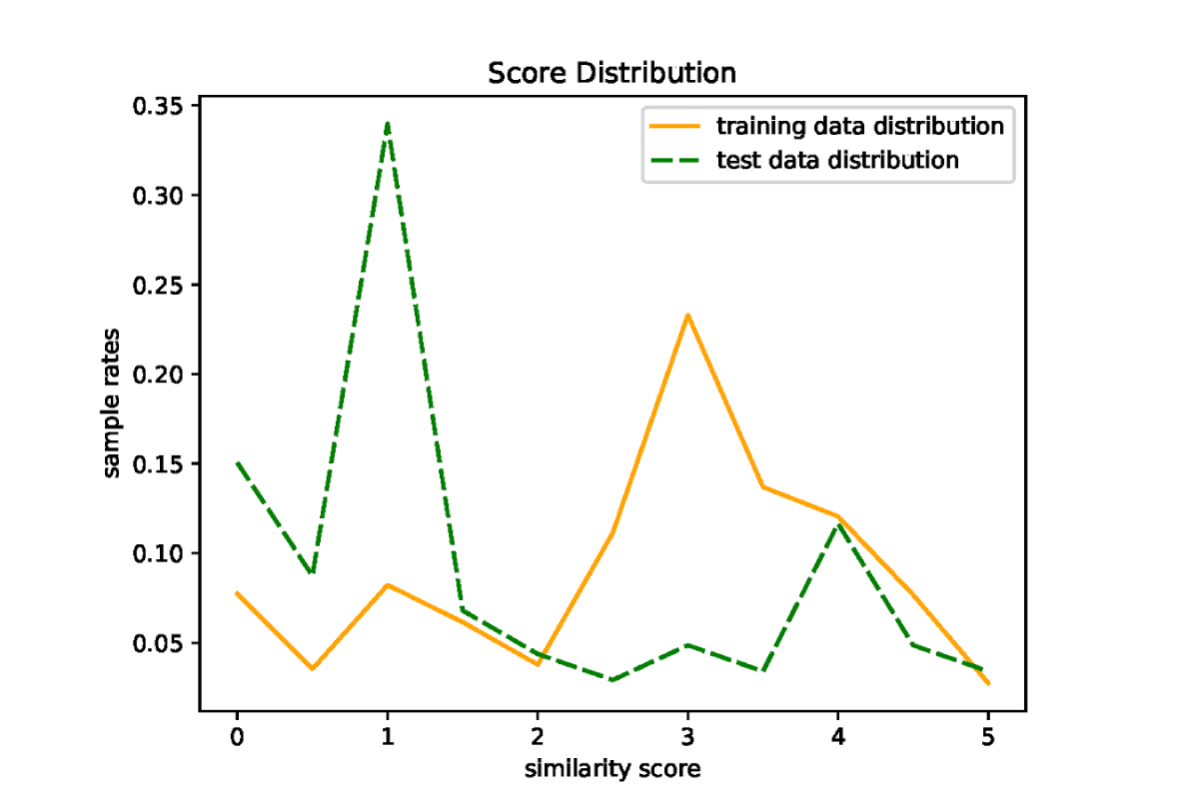
Similarity interval distribution in the training and test data sets.

The first type of error is related to polarity of clinical text snippets as our model is insensitive to positive and negative words. For example, as shown in Table 4, because both clinical text snippets in example 1 depict coughing up, their STS score predicted by our model is 2.5, but their gold-standard STS score is 1.0 as the polarity of the first text snippet is positive, whereas that of the second text snippet is negative. The second type of error is related to prescriptions that include medication names, usages, and dosages. For example, the gold-standard STS score of example 2 in Table 4 is 1.0 as the medications in the 2 text snippets are completely different, but the STS score of the example predicted by our model is 2.5 as some other words are the same in the 2 text snippets. Just because our model cannot extract medical information comprehensively, there are lots of errors of the second type. For further improvement, we need a comprehensive information extraction module to extract polarity information and medications with usage and dosage attributes besides the current 9 kinds of clinical entities. A possible way is to integrate the existing tools specifically for polarity information extraction (such as SenticNet [47]) or medication extraction (such as MedEx [48]) into our model. We also find that the scores of mispredictions are close to 2.5, which may be caused by the different STS score distributions of the training and test sets. As shown in Figure 3, the STS scores of most sentence pairs in the training set concentrate in [2.5, 3.5], whereas those in the test set concentrate in [0.5, 1.5]. The difference is remarkable. It is reasonable to obtain the STS scores of mispredictions around the average score of the training set.

**Table 4:** Examples of errors on the test set.

Number	Example
1	*Sentence 1:respiratory: positive for coughing up mucus (phlegm), dyspnea and wheezing*. *Sentence 2: negative for coughing up blood and dry cough*. Gold-standard: 1.0 Predicted: 2.5
2	*Sentence 1: ibuprofen [motrin] 800 mg tablet 1 tablet by mouth four time a day as needed.* *Sentence 2: lisinopril 10 mg tablet 1 tablet by mouth one time daily.* Gold-standard: 1.0 Predict: 2.4

### Effect of Entity-Level Representation

Although the results in Table 3 show that any one of the 2 entity-level representations enhances the BERT-based model, some limitations also exist. In the case of entity I, we only consider type semantic information, but no entity semantic information. In the case of entity II, only about 20% (220/1080) of clinical entities recognized by cTAKES [42] can be mapped to Mesh via dictionary look-up. There are 2 directions for improvement: (1) introduce entity semantic information into entity I, and (2) improve entity mapping performance in entity II and find a larger KG instead of Mesh.

### Conclusions

In this paper, we propose an enhanced BERT-based model for ClinicalSTS by introducing a character-level representation and an entity-level representation. Experiments on the 2019 n2c2/OHNLP track on ClinicalSTS in 2019 indicate that both the character-level representation and the entity-level representation can enhance the BERT-based ClinicalSTS model, and our enhanced BERT-based model achieves competitive performance with other state-of-the-art models. In addition, domain-specific pretrained BERT models are better than general pretrained BERT models.

## Abbreviations

BERT: bidirectional encoder representation from transformers
ClinicalSTS: clinical semantic textual similarity
CNN: convolutional neural network
EHR: electronic health record
KG: knowledge graph
MLP: multilayer perceptron
NLP: natural language processing
OHNLP: Open Health Natural Language Processing
OOV: out of vocabulary
PCC: Pearson correlation coefficient
SemEval: Semantic Evaluation
STS: semantic textual similarity
